# Protein-bound uremic toxins impaired mitochondrial dynamics and functions

**DOI:** 10.18632/oncotarget.20773

**Published:** 2017-09-08

**Authors:** Chiao-Yin Sun, Mei-Ling Cheng, Heng-Chih Pan, Jia-Hung Lee, Chin-Chan Lee

**Affiliations:** ^1^ Department of Nephrology, Chang Gung Memorial Hospital, Keelung, Taiwan; ^2^ Kidney Research Center, Chang Gung Memorial Hospital, Taoyuan, Taiwan; ^3^ School of Medicine, Chang Gung University, Taoyuan, Taiwan; ^4^ Metabolomics Core Laboratory, Healthy Aging Research Center, Chang Gung University, Taoyuan, Taiwan; ^5^ Clinical Phenome Center, Chang Gung Memorial Hospital, Taoyuan, Taiwan; ^6^ Department of Biomedical Sciences, College of Medicine, Chang Gung University, Taoyuan, Taiwan; ^7^ Medical Research Center, Chang Gung Memorial Hospital, Keelung, Taiwan

**Keywords:** uremic toxins, metabolic stress, mitochondrial fusion, mitochondrial mass, mitophagy

## Abstract

Protein-bound uremic toxins, indoxyl sulfate and *p*-cresol sulfate, increase oxidative stress and adversely affect chronic kidney disease progression and cardiovascular complications. In this study, we examined whether mitochondria are the target of indoxyl sulfate and *p*-cresol sulfate intoxication *in vivo* and *in vitro*. The kidneys of 10-week-old male B-6 mice with ½-nephrectomy treated with indoxyl sulfate and *p*-cresol sulfate were used for the animal study. Cultured human renal tubular cells were used for the *in vitro* study. Our results indicated that indoxyl sulfate and *p*-cresol sulfate impaired aerobic and anaerobic metabolism *in vivo* and *in vitro*. Indoxyl sulfate and *p*-cresol sulfate caused mitochondrial fission by modulating the expression of mitochondrial fission–fusion proteins. Mitochondrial dysfunction and impaired biogenesis could be protected by treatment with antioxidants. The *in vitro* study also demonstrated that indoxyl sulfate and *p*-cresol sulfate reduced mitochondrial mass by activating autophagic machinery. In summary, our study suggests that mitochondrial injury is one of the major pathological mechanisms for uremic intoxication, which is related to chronic kidney disease and its complications.

## INTRODUCTION

Impairment of mitochondrial function has been associated with numerous pathological conditions and aging [1, 2]. Mitochondria and oxidative stress were important contributors in the renal inflammatory process. Deregulation of the mitochondrial respiratory mechanism has been described in patients with chronic renal disease associated with increased oxidative stress [3]. Mitochondria are highly dynamic organelles that constantly undergo fission and fusion. The balance of mitochondrial dynamics is crucial for controlling mitochondrial quality and cellular metabolism [4]. In diabetic nephropathy, high glucose levels can cause the fragmentation of mitochondria and induce the excess production of reactive oxygen species (ROS). In addition, electron transport chain defects, which result in electron leakage and superoxide radical formation, have been established in diabetic nephropathy [5, 6].

Mitophagy, in which damaged mitochondria are selectively removed, is considered an adaptive response to stress that promotes cell survival under stress. Mice with impaired mitophagic turnover in podocytes and the tubular epithelium exhibit renal histological changes similar to human idiopathic focal segmental glomerulosclerosis [7]. Increased oxidative stress, mitochondrial fragmentation, and dysfunctional mitochondria accumulation are observed in the tubules of diabetic mice [8]. Transforming growth factor (TGF)-β is a major profibrogenic cytokine for renal cell injury in chronic kidney disease (CKD) [9]. TGF-β induces both renal cell apoptosis and renal fibrosis. TGF-β is also associated with mitochondrial dysfunction and fragmentation in multiple renal cells [10]. Mitochondria are the main organelles producing ROS, which play key roles in the initiation and modulation of cell death [11]. Disrupting mitochondria-derived ROS production may attenuate TGF-β-induced fibrosis [12].

Protein-bound uremic toxins, indoxyl sulfate (IS) and *p*-cresol sulfate (PCS), adversely affect CKD progression and its complications [13]. Cross talk between mitochondria and reduced nicotinamide adenine dinucleotide phosphate (NADPH) oxidases play a key role in the maintenance of the cellular redox status [14]. IS and PCS stimulate intracellular and extracellular ROS production through a pathway involving an NADPH oxidase or an NADPH-like oxidase [15–17]. In addition to oxidative stress, IS and PCS induce inflammatory reactions and activate the renin–angiotension–aldosterone system (RAAS), which is associated with renal fibrosis [18, 19]. Inflammation causes mitochondrial stress by inducing drastic metabolic changes associated with alterations in mitochondrial dynamics that shift the balance between aerobic glycolysis and oxidative phosphorylation [20]. RAAS activation not only alters cellular metabolism but also induces mitochondrial production of ROS, which function as pathological signals [21, 22]. Recent evidence has indicated that IS can impair mitochondrial function and biogenesis by increasing mitochondrial depolarization and decreasing mitochondrial mass *in vitro*[23]. Treatment with a uremic toxin binder can normalize citrate synthetase activity, mitochondrial biogenesis, and superoxide production in the skeletal muscle of CKD mice [24]. Here, we report that IS and PCS deregulate mitochondrial metabolism, mass, and dynamics.

## RESULTS

### Identification of deregulated mitochondrial dynamics by indoxyl sulfate and *p*-cresol sulfate in study mice

**Figure 1:**
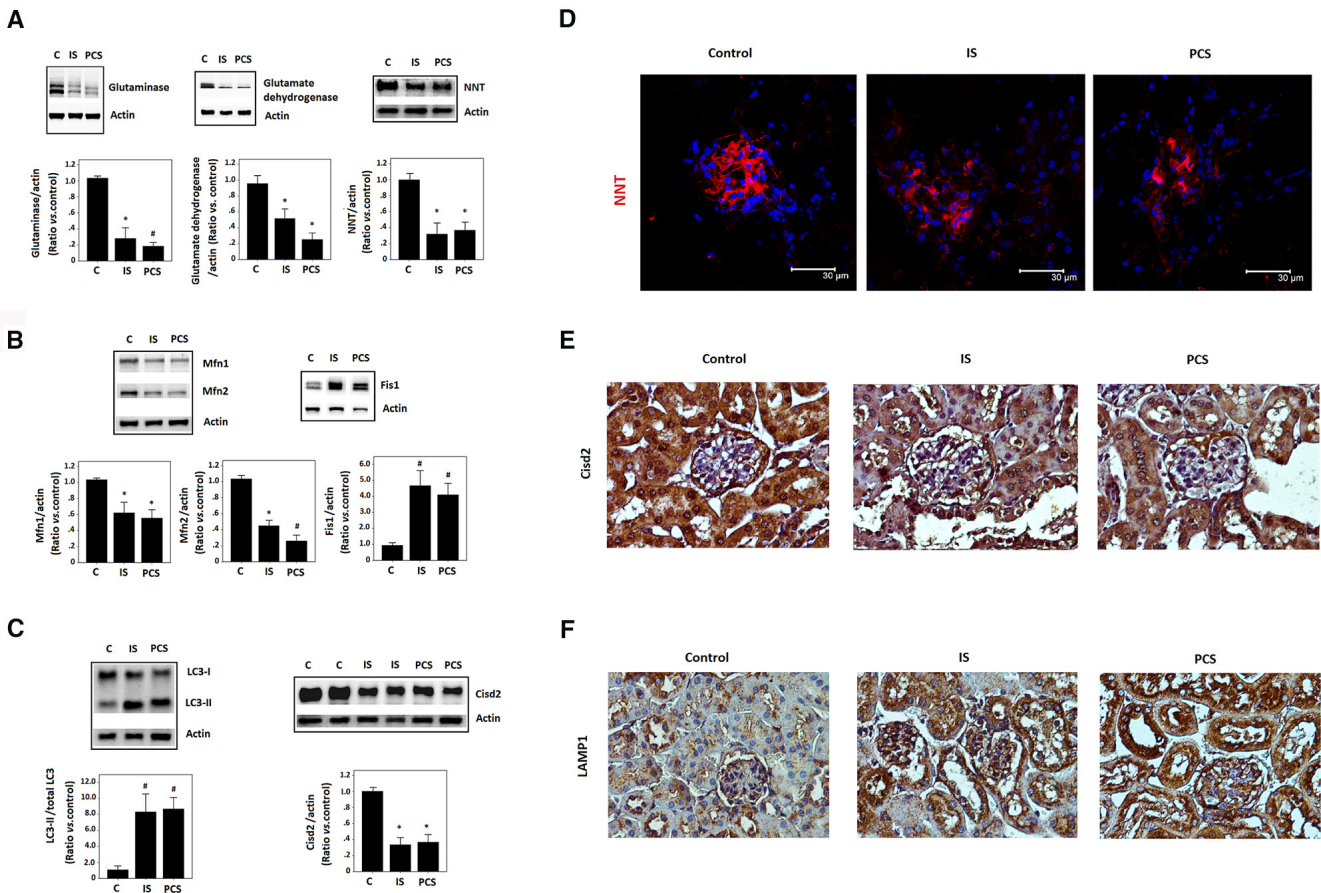
Indoxyl sulfate and *p*-cresol sulfate impaired mitochondrial dynamics *in vivo* Mice with ½-nephrectomy treated with IS or PCS for 1 week were used for this study (experimental group, n=8). The control mice with ½-nephrectomy received normal saline at the same volume for 1 week (control group, n=8). **(A)** Western blotting (cropped blots) results for glutaminase, glutamate dehydrogenase, and NNT. **(B)** Western blotting (cropped blots) results Mfn1, Mfn2, and Fis1. **(C)** Western blotting (cropped blots) results for LC3 andCisd2. **(D)** Representative results of immunofleurocent staining for NNT. **(E)** Representative results of immunohistological staining for Cisd2. **(F)** Representative results of immunohistological staining for LAMP1 (combined results shown as mean±SD). (C: control; IS: indoxyl sulfate; PCS: *p*-cresol sulfate) (*P< 0.05; #*P*< 0.01, *vs.* control ) (microscopy: 400×).

To identify the possible mitochondrial dysfunction induced by IS and PCS *in vivo*, mice with ½-nephrectomy that were treated with IS or PCS were evaluated in this study. Mitochondrial glutaminase and glutamate dehydrogenase are the key enzymes in nitrogen metabolism and the acid–base balance [25]. Our results showed that IS and PCS treatment significantly reduced glutaminase and glutamate dehydrogenase protein levels in study mice (Figure 1A). Western blotting results also demonstrated that IS and PCS treatment significantly reduced nicotinamide nucleotide transhydrogenase (NNT) protein expression (Figure 1A). NNT is a mitochondrial inner membrane marker that catalyzes the hydride transfer of reducing equivalent between NAD(H) and NADP(+) in a reaction coupled to proton translocation across the inner mitochondrial membrane; NNT plays a crucial role in mitochondrial antioxidant defense [26]. These results suggest that IS and PCS impair renal mitochondrial function *in vivo*.

To investigate the putative impact of uremic toxins on mitochondrial dynamics, Western blotting of the regulators of mitochondrial morphology, namely mitofusin1 (Mfn1), Mfn2, and mitochondrial fission 1(Fis1), was conducted. IS and PCS significantly reduced Mfn1 and Mfn2 but increased Fis1 protein expression in the kidneys of study mice (Figure 1B). In addition, increased modified microtubule-associated protein 1 light chain 3 II (LC3-II), which is a regulator of autophagy, was noted in the kidneys of study mice treated with IS and PCS (Figure 1C). Cisd2, another regulator of autophagy, was also downregulated by IS and PCS *in vivo* (Figure 1C). Immunofleurocent staining results that the intensity of NNT decreased significantly in the glomerulus of the kidneys of mice treated with IS and PCS (Figure 1D). Cisd2 plays key roles in maintaining mitochondrial integrity [27]. Immunohistological staining showed that Cisd2 expression was high in the renal tubules of the control mice. The staining intensity of Cisd2 decreased significantly in the renal tubules of study mice treated with IS and PCS (Figure 1E). The positive staining for the lysosome marker lysosomal-associated membrane protein 1 (LAMP1) was also increased in the renal tubules of mice treated with IS and PCS (Figure 1F). These results suggest that IS and PCS impair mitochondrial dynamics and induce autophagy *in vivo*.

### Cellular metabolic changes in cells treated with indoxyl sulfate and *p*-cresol sulfate

**Figure 2:**
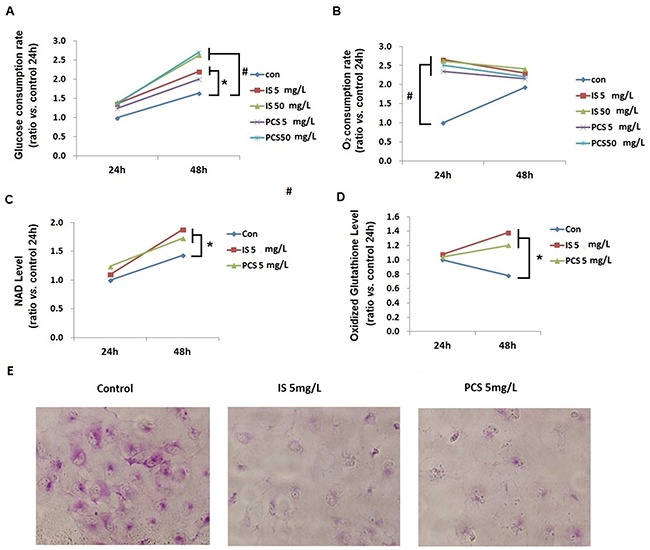
Indoxyl sulfate and *p*-cresol sulfate increased cellular catabolism and oxidized metabolite production *in vitro* Cultured human renal tubular cells (HK2) treated with IS or PCS under the serum-free condition for 24 and 48h were analyzed. Control cells were cultured under the serum-free condition only. The concentrations of IS and PCS for this study are indicated in the figures. Cellular levels of NAD and oxidized glutathione were measured using LC/MS/MS. The mean relative ratios verses control cells are plotted. The reactions for the experiments for A, B, C, and D are repeated in triplets, and the mean values are plotted. **(A)** glucose consumption rate; **(B)** oxygen consumption rate; **(C)** NAD levels; **(D)** oxidized glutathione levels; **(E)** HK2 cells were stained with PAS after IS and PCS treatment for 24h to detect glycogen storage. Each reaction was repeated in triplet, and the mean values were showed in the plots. (con: control; IS: indoxyl sulfate; PCS: *p*-cresol sulfate) ( *P< 0.05; #*P*< 0.0, *vs.* control) (microscopy: 200×).

To study the metabolic impact of IS and PCS on cellular metabolism, cultured HK2 cells treated with IS and PCS were analyzed. The oxygen and glucose consumption rates were calculated by detecting the decrease in oxygen and glucose concentrations in medium. Our results showed that the oxygen consumption rates increased significantly after 24h treatment with IS or PCS, but this finding was not persistently noted after 48h treatment with IS or PCS. By contrast, the significant increase in glucose consumption rates was not observed until after 48h treatment with IS or PCS (Figure 2A, 2B). Metabolite analysis demonstrated that the cellular levels of oxidized metabolites, NAD and oxidized glutathione, significantly increased after 48h treatment with IS and PCS (Figure 2C, 2D). After 24h treatment with IS and PCS, periodic acid–Schiff (PAS) staining showed lower PAS staining intensity in cells treated with IS and PCS than in control cells (Figure 2E). This finding indicated that glycogen storage decreased in cells treated with IS and PCS.

### Indoxyl sulfate and *p*-cresol sulfate attenuated cellular energy pool

**Figure 3:**
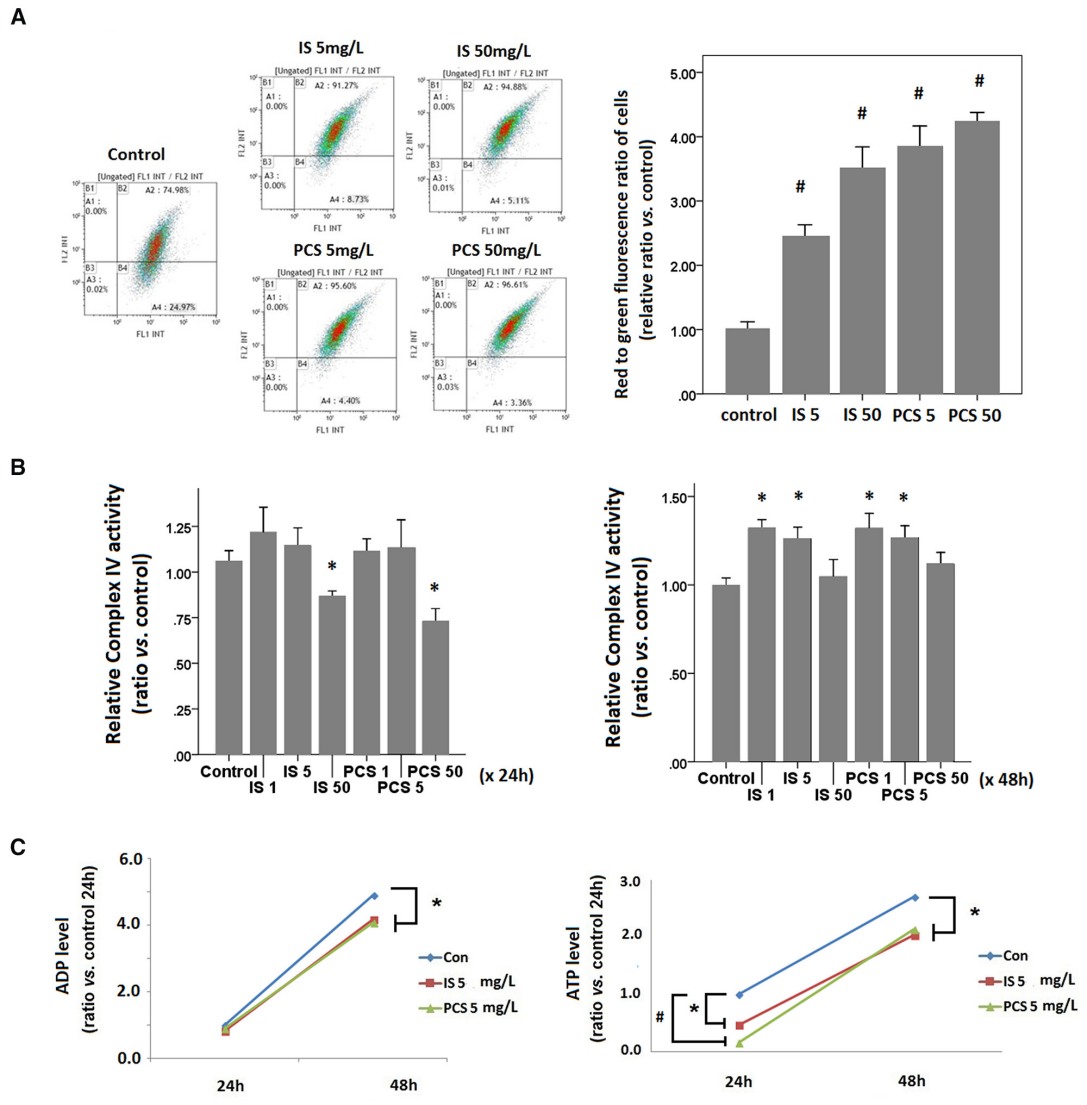
Indoxyl sulfate and *p*-cresol sulfate increased energy consumption *in vitro* Cultured human renal tubular cells (HK2) were stained with JC-1 dye after treatment with IS and PCS at the concentrations of 5 and 50 mg/L for 24h. Each reaction for flow cytometry, cytochrome complex IV activity, and metabolite analysis was repeated in triplet. The mean values were then plotted. The IS and PCS concentrations and treatment duration for B and C are shown in the figure. **(A)** Flowcytometry results of JC-1 staining, and plotting for the relative red to green fluorescence ratios of cells; **(B)** results of cytochrome complex IV activity of HK2 cells; **(C)** cellular levels of ADP and ATP. Each reaction was repeated in triplet and the results were shown as mean±SD in plots. (con: control; IS: indoxyl sulfate; PCS: *p*-cresol sulfate) (*P< 0.05; #*P*< 0.01, *vs.* control).

Flow cytometry results showed that HK2 cells treated with IS and PCS for 24h exhibited significantly increased red to green fluorescence among cells with JC-1 staining. This result indicated that HK2 cells treated with IS and PCS had higher mitochondrial membrane potential than control cells (Figure 3A). The cytochrome complex IV activity assay was performed with isolated mitochondria. The complex IV activity of cells treated with IS and PCS at concentrations of 1 and 5 mg/L for 24h did not significantly differ from that of control cells; however, treatment with a high concentration of IS and PCS (50 mg/L) attenuated complex IV activity (Figure 3B) However, the complex IV activity of cells treated with IS and PCS at concentrations of 1 and 5 mg/L for 48h was significantly higher than that of control cells (Figure 3B).

IS and PCS increased glucose and oxygen consumption, mitochondrial membrane potential, and complex IV activity. These results suggest that IS and PCS stimulate aerobic respiration in residual mitochondria. Although aerobic respiration was increased in residual mitochondria, the cellular adenosine diphosphate and adenosine triphosphate levels were significantly lower than those in control cells (Figure 3C). This result suggests that IS and PCS attenuates the cellular energy pool by increasing energy consumption.

### Indoxyl sulfate and *p*-cresol sulfate enhanced mitochondrial fission *in vitro*

**Figure 4:**
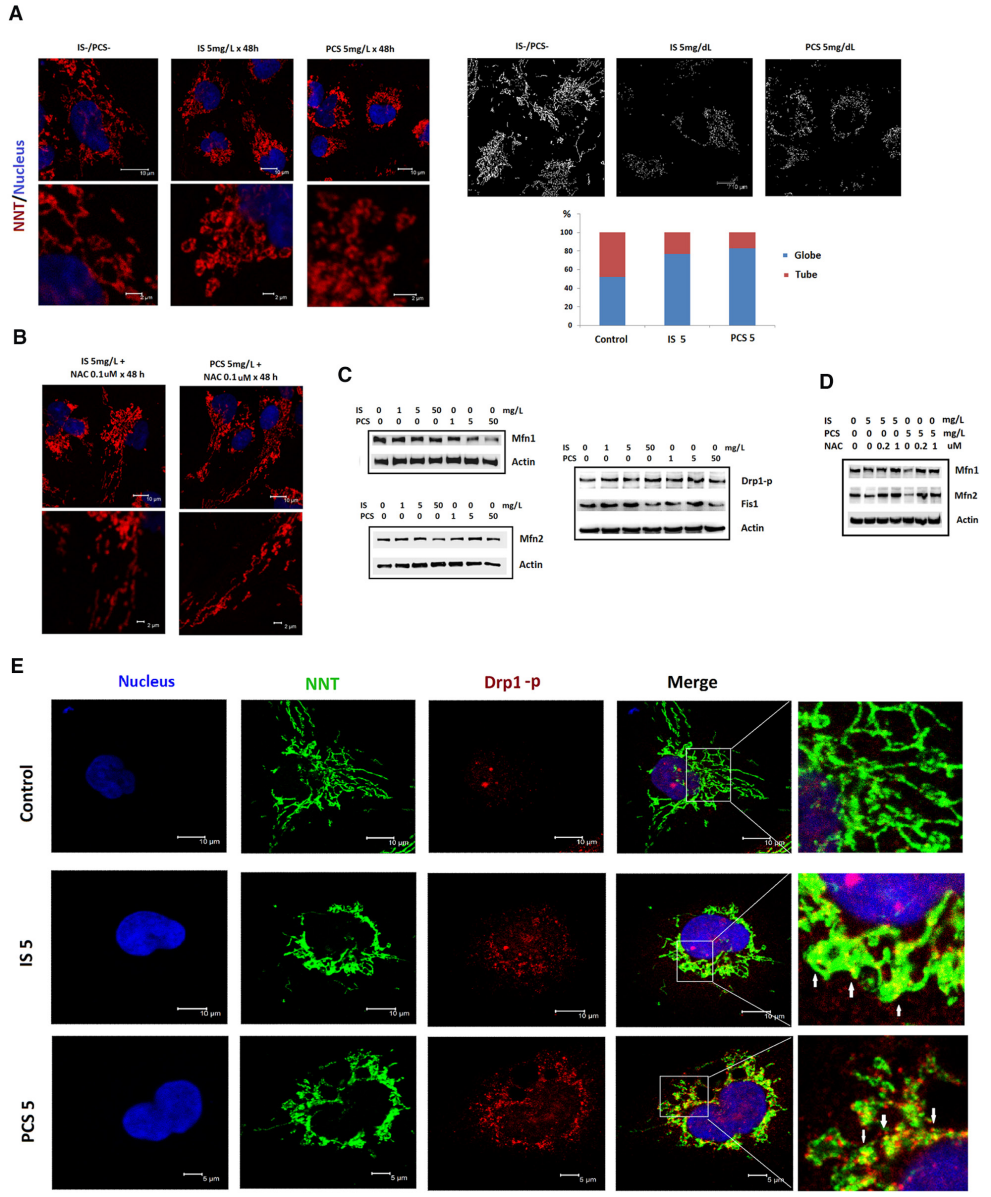
Indoxyl sulfate and *p*-cresol sulfate altered mitochondria morphological dynamics *in vitro* Cultured human renal tubular cells (HK2) were treated with IS and PCS for 48h under the serum-free condition. Mitochondria morphology was analyzed by immunostaining for NNT. For the antioxidation study, the HK2 cells were pretreated with NAC for 2 h before IS and PCS treatment. The control cells were cultured under the serum-free condition. The concentrations of IS, PCS, and NAC for each study are shown in the figure. **(A)** Representative results of immunofluorescent staining against NNT in control and IS and PCS-treatment cells; the mitochondria with globular and donut shapes were categorized as globular form. The mitochondria with simple, twisting and branching tubular shapes were categorized as tubular form. The average percentage of globular and tubular mitochondria of control and IS/PCS-treated cells were plotted. **(B)** Representative results of immunofluorescent staining against NNT in IS and PCS-treated cells with NAC pretreatment. **(C)** Western blotting (cropped blots) results forMfn1, Mfn2, Drp1-p, and Fis1in control and IS and PCS-treated cells. **(D)** Western blotting (cropped blots) results for Mfn1 and Mfn2 in IS and PCS-treated cells with or without NAC pretreatment. **(E)** Representative confocal microscopy images of costaining with anti-NNT and anti-Drp1-p. The colocalization of NNT and Drp1 signals is indicated by a white arrow. (IS: indoxyl sulfate; PCS: *p*-cresol sulfate) (confocal microscopy: 400×).

To study the putative effects of IS and PCS on mitochondrial dynamics, immunofluorescent staining of NNT was conducted. The results showed that IS and PCS significantly increased the globular form of mitochondria (Figure 4A). Pretreatment with NAC could reverse the mitochondrial dynamic changes caused by IS and PCS (Figure 4B). Western blotting results showed that IS and PCS downregulated Mfn1 and Mfn2, which regulate mitochondrial fusion, in a dose-dependent manner. By contrast, IS and PCS increased the levels of phosphorylated Drp1, which mediates mitochondrial fission (Figure 4C). Fis1 is also a signal protein involved in mitochondrial fission. This study showed that low IS and PCS doses (1 and 5 mg/L) upregulated Fis1 levels *in vitro*. However, high IS and PCS doses downregulated Fis1 levels in cultured HK2 cells (Figure 4C). Pretreatment with NAC reversed the downregulated Mfn1 and Mfn2 levels induced by IS and PCS (Figure 4D). Costaining with phosphorylated Drp1 and NNT demonstrated that IS and PCS treatment not only increased the phosphorylated Drp1 level but also recruited the phosphorylated Drp1 to mitochondria (Figure 4E).

### Indoxyl sulfate and *p*-cresol sulfate induced mitophagy in cultured renal tubular cells

**Figure 5:**
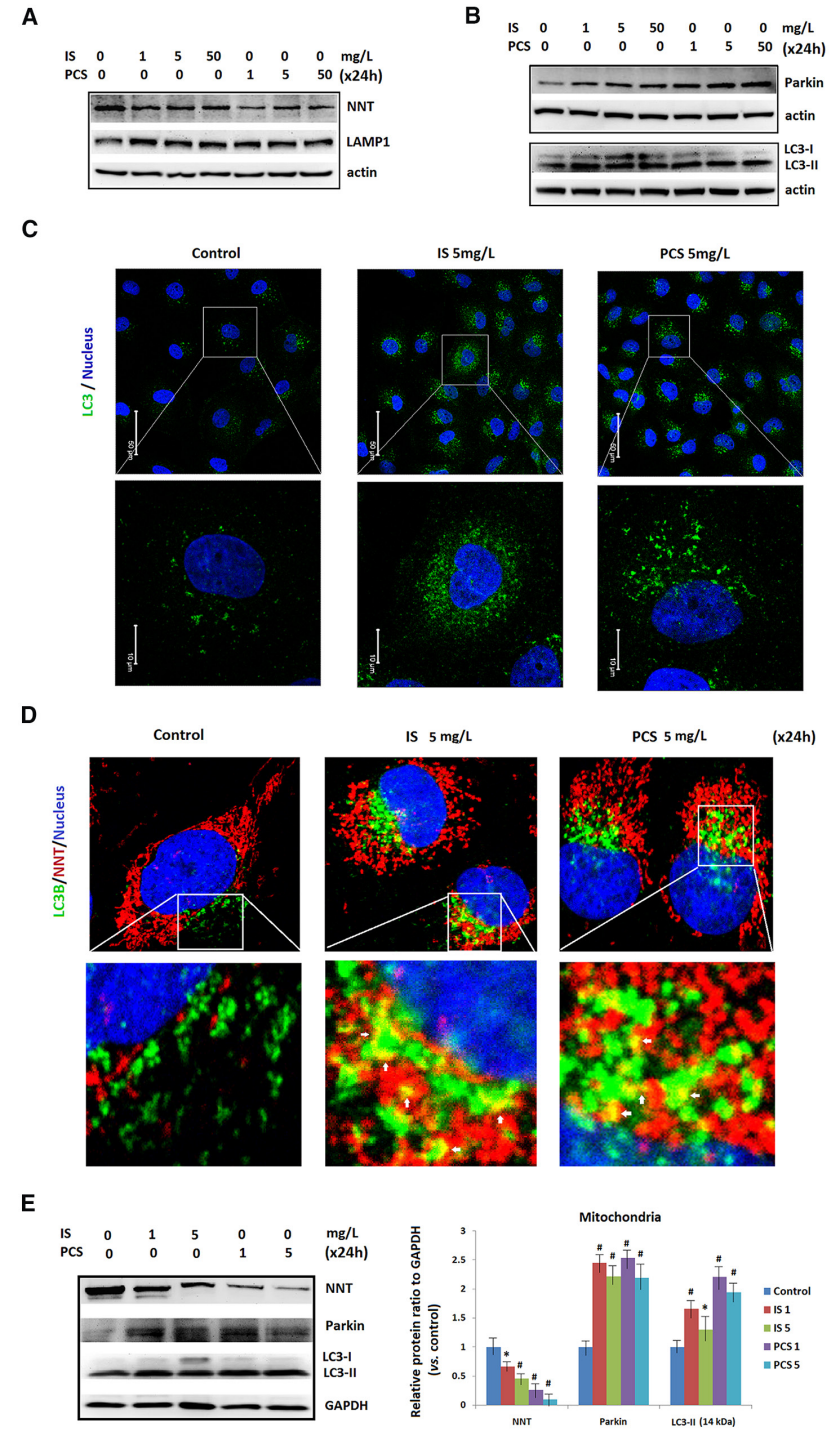
Indoxyl sulfate and *p*-cresol sulfate induced autophagy in mitochondria Cultured human renal tubular cells (HK2) were treated with IS and PCS for 24h under the serum-free condition. The control cells were cultured under the serum-free condition. **(A)** Western blotting (cropped blots) results for NNT and LAMP1. **(B)** Western blotting (cropped blots) results for Parkin and LC3. **(C)** Representative results of LC3 puncta formation of HK2 cells treated with IS and PCS were showed. The concentrations of IS and PCS were indicated as figure legends. **(D)** Representative confocal microscopy images of immunofluorescent staining with LC3.The colocalization of NNT and LC3 signals is indicated by a white arrow. **(E)** Western blotting results for NNT, Parkin and LC3 with isolated mitochondria from cells treated with IS and PCS for 24 h. GAPDH was used as internal control for Western blotting. Each reaction was repeated in triplet, and the average protein ratios to the GAPDH verse control cells were plotted. Each reaction was repeated in triplet, and the results were shown as mean±SD in plots. (IS: indoxyl sulfate; PCS: *p*-cresol sulfate) (*P< 0.05; #*P*< 0.01, *vs.* control) (confocal microscopy: 400×).

Autophagy was a key protective mechanism for cell survival under stress. [28] Western blotting results revealed that NNT was downregulated in HK2 cells after 24h treatment with IS and PCS (Figure 5A). This finding suggests that IS and PCS reduces mitochondrial mass *in vitro*. This study also showed that LC3-II, LAMP1 and Parkin were upregulated in cells treated with IS and PCS (Figure 5A, 5B). Parkin, an E3 ligase, mediates the selective removal of damaged mitochondria through mitophagy [29].The immunostaining results for LC3 revealed that IS and PCS treatment significantly increased LC3 puncta formation in HK2 cells (Figure 5C). In addition, overlapping of NNT and LC3 signals increased in cells treated with IS and PCS (Figure 5D). Western blotting with protein extracts of isolated mitochondria from cells treated with IS and PCS were performed to define the mitochondrial NNT, Parkin and LC3 expression. The results indicated that IS-and PCS-treated cells had increased mitochondrial Parkin and LC3-II, but decreased mitochondrial NNT expression (Figure 5E). To detect the mitochondrial injury, we also performed the immunofleurocent staining with the autophagy markers, LAMP1 and Rab11 with cultured renal tubular cells treated with IS and PCS. The results indicated that mitochondrial localization of LAMP1 and Rab11 increased in cells treated with the indoxyl sulfate and p-cresol sulfate (Supplementary Figure 1). These aforementioned results suggest that IS and PCS can reduce mitochondrial mass through autophagic machinery.

**Figure 6:**
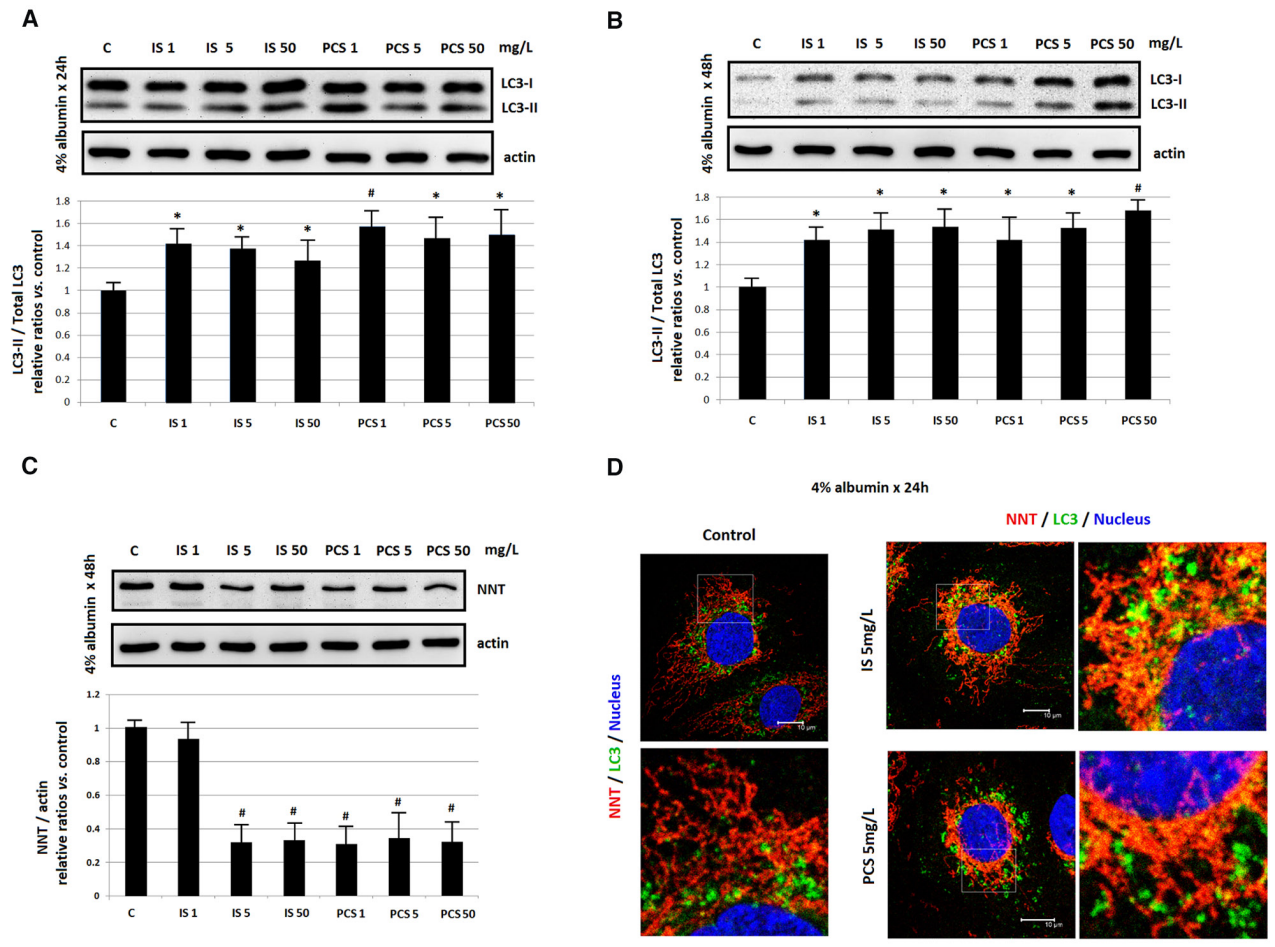
Mitochondrial damage by indoxyl sulfate and *p*-cresol sulfate in the presence of albumin *in vitro* Cultured HK2 cells were treated with IS and PCS under the serum-free medium which containing 4 % albumin. The concentrations of IS and PCS and treatment duration for the study were indicated as figure legends. **(A)**, **(B)** Western blotting results for LC3; **(C)** Western blotting results for NNT; **(D)** represent immunofleurocent staining results for NNT and LC3. Each reaction was repeated in triplet, and the results were shown as mean±SD in plots. (C: control; IS: indoxyl sulfate; PCS: *p*-cresol sulfate) (*P< 0.05; #*P*< 0.01, *vs.* control) (confocal microscopy: 400×).

For IS and PCS are protein-bound uremic toxins, the *in vitro* study in the presence of 4% albumin were performed. The Western blotting results showed that IS and PCS treatment could activate LC3 (Figure 6A, 6B) and attenuate NNT level (Figure 6C) in the culture condition with albumin. The immunostaining results also revealed that IS and PCS significantly increased the co-localization of NNT and LC3 in the presence of albumin (Figure 6D).

## DISCUSSION

The main findings of this study indicate that IS and PCS induce mitochondrial toxicity. This study found that IS and PCS not only affected aerobic respiration but also anaerobic respiration. Our study indicated that IS and PCS caused mitochondrial injury by inducing oxidative and metabolic stress. IS and PCS significantly increased the expression of mitofission proteins but reduced the expression of mitofusion proteins. In addition, IS and PCS treatment significantly increased the factors of mitophagy, activated LC3, Parkin, and LAMP1, *in vitro* and *in vivo*. This result suggests that IS and PCS induces mitochondrial dynamic changes by causing metabolic stress, which can further decrease mitochondrial mass through mitophagy.

Mitochondrial oxidative stress is a major cause of mitochondrial fragmentation. It has been demonstrated that IS and PCS induces oxidative stress by activating NADPH oxidase [16, 17]. Our study showed that decreasing oxidative stress caused by IS and PCS by NAC significantly reduced the expression of the mitofission proteins Drp1 and Fis1, suppressing mitofission. A recent study in human umbilical vein endothelial cells (HUVECs) also showed similar results. In HUVECs, treatment with antioxidants, vitamin C and NAC, could prevent mitochondrial fragmentation induced by IS [23].

Mitophagy is crucial for controlling mitochondrial quality and biogenesis [30]. It has been reported that IS can reduce mitochondrial mass *in vitro*[23]. The current study demonstrated that IS and PCS might activate mitophagy, which selectively eliminates mitochondria. Our results indicated that IS and PCS activated autophagic mechanisms. Our study showed that autophagic machinery (LC3 puncta) colocalized with a mitochondrial marker (NNT) under the cytotoxic stress caused by IS and PCS. Parkin-mediated selective mitophagy pathway has been suggested to function as an inducible stress-response mechanism. Recruitment of Parkin to the mitochondria is critical for subsequent mitophagy [30, 31]. This study found that the expression of Parkin increased as the IS and PCS concentration increased *in vitro*. Although mitophagy eliminates damaged mitochondria and mitochondrial biogenesis generates new mitochondria, mitochondrial mass decreased when the damage beyond cellular compensation.

Cisd2 is the causative gene for the degenerative disease of Wolfram syndrome 2 [32]. Cisd2 protein exerts an antiaging function by preventing mitochondrial degeneration [33, 34]. Cisd2 depletion causes mitochondrial breakdown and dysfunction accompanied by autophagy. Cisd2 can antagonize BECN1-mediated cellular autophagy by modulating calcium homeostasis at the endoplasmic reticulum [35, 36]. In this study, IS and PCS toxicity decreased Cisd2 expression *in vivo*. However, the protective roles of Cisd2 in the kidney injury induced by IS and PCS were still not defined.

In conclusion, IS and PCS might increase mitochondrial oxidative stress, which causes mitochondrial fission by modulating the expression of mitochondrial fission–fusion proteins and activating autophagic machinery. Decompensated mitophagy can reduce mitochondrial mass and lead to deregulated cellular metabolism. Our study suggests that mitochondrial injury is one of the major pathological mechanisms for uremic intoxication, which is related to CKD and its complications.

## MATERIALS AND METHODS

### Study approval

All animal experiments were approved by the Institutional Animal Care and Use Committee of Chang Gung Memorial Hospital (IACUC number: 2008101703, 2012010903). Our animal center is an AAALAC certified center and this study was performed in accordance with all the relevant guidelines and regulations.

### Animal model

The animal model was modified from our previous study [18]. In brief, 10-week-old male B-6 mice with ½-nephrectomy were used in this study. The study animals were divided into control and experimental groups. The control mice (n = 8) received daily phosphate-buffered saline (PBS) injections at the same volume as the experimental mice for 1 week. The experimental mice received intraperitoneal injection with IS (Sigma-Aldrich, St. Louis, MO) (n = 8) or PCS (Kureha Corporation, Tokyo, Japan) (n = 8) at a dose of 100 mg/kg/day for 1 week. The kidneys of study animal were harvested for following study.

### Cell culture

Human renal tubular cells (HK2) were cultured in the DMEM based medium as described previously. HK2 cell cultures at approximately 70% confluence were synchronized under serum-free conditions for 48 h. These cells were then treated with IS or PCS at concentrations of 0, 1, 5, and 50 mg/L under the serum-free condition in the presence or absence of 4 % albumin (Sigma-Aldrich). For the antioxidation study, the HK2 cells were pretreated with *N*-acetylcysteine (NAC) (Sigma-Aldrich) for 2 h before IS and PCS treatment under the serum-free condition. The concentrations of IS, PCS, and NAC and the treatment duration are illustrated in figure legends.

### Western blotting

Total protein was extracted using a commercial kit according to the manufacturer's instructions (Protein Extraction Kit, Millipore, Billerica, MA). Thirty micrograms of protein from each sample was mixed with sample loading buffer and loaded onto separate lanes on a 12%sodium dodecyl sulfate-polyacrylamide gel. Proteins were electrotransferred onto polyvinylidene fluoride membranes (0.2 μm: Immun-Blot, Bio-Rad, Hercules, CA) and then immunoblotted with primary antibodies. The intensity of each band was quantified using NIH Image software (Bethesda, MD), and the densitometric intensity corresponding to each band was normalized against β-actin expression.

### Immunostaining and periodic acid–Schiff stain

Paraffin tissue sections were cut, mounted, deparaffinized, rehydrated, and stained with hematoxylin–eosin by using standard histological techniques. For immunohistochemical staining, the Ventana Benchmark automated staining system and Ventana reagents were used (Ventana Medical Systems, Tucson, AZ), and primary antibodies. The tissue sections were observed using light microscopy (Nikon Eclipse Ti, Tokyo, Japan). For immunofluorescence staining, cells were incubated with a primary antibody, followed by incubation with a fluorescent secondary antibody. The sections were counterstained with 4′,6-diamidino-2-phenylindole (1:500 dilution; Sigma-Aldrich) to identify cellular nuclei. The stained samples were observed under a confocal microscope (Leica Microsystems, Bannockburn Ill, Wetzlar, Germany). The mitochondrial morphology was analyzed by MicroP software with the default setting [37]. The antibodies used for Western blot and immunostaining were listed in Supplementary Table 1. For detecting cellular glycogen storage, HK2 cells were stained with a PAS staining system (Sigma-Aldrich) according to the product instructions. The cells were fixed in formalin-ethanol fixative solution, followed by staining with periodic acid solution and Schiff's reagent. The air dried slides were examined microscopically (400×) (Nikon Eclipse Ti).

### Mitochondrial isolation and complex IV enzyme activity

Mitochondria were isolated from HK-2 cells by using the Qproteome mitochondrial isolation kit (Qiagen, Venlo, Netherlands) according to the standard protocol. In brief, washed cells were first suspended in lysis buffer; subsequently, the cells were centrifuged to obtain a pellet consisting of compartmentalized organelles. The resulting pellet was resuspended in disruption buffer, repeatedly passed through a narrow-gauge needle, and centrifuged to obtain a pellet consisting of nuclei, cell debris, and unbroken cells. The supernatant, which contained mitochondria and the microsomal fraction, was re-centrifuged to obtain a pellet consisting of mitochondria. After removal of the supernatant, mitochondria were washed and resuspended in mitochondria storage buffer.

Freshly isolated mitochondria were used for assaying complex IV enzyme activity. The concentrations of mitochondrial proteins were determined using the Pierce^TM^ BCA protein assay (Pierce Biotechnology, Waltham, MA). Freshly isolated mitochondria were maintained on ice until use in subsequent assays. Mitochondrial complex IV enzyme activity was determined using a mitochondrial complex IV enzyme activity assay kit (Abcam, Cambridge, MA) according to the manufacturer's protocol. The sample at a concentration of 5.0 mg/mL was analyzed. Complex IV was immunocaptured within the wells, and its activity was determined calorimetrically at OD 550 nm after the oxidation of reduced cytochrome C. To determine the activity in the sample, the slope was calculated using the formula: Rate = (Absorbance 1 − Absorbance 2)/Time (min). The relative complex IV activity was obtained by comparing the sample rate with the rate of the control (normal) sample and with the rate of the null (background). In this study, each reaction was repeated in triplet. The relative ratios versus control were plotted.

### Oxygen consumption

The oxygen consumption of HK2 cells was determined using a Clark-type polarographic electrode (Mitocell Respirometery SystemMT200/MT200A, Strathkelvin Instruments, North Lanarkshire, Scotland) according to the manufacturer's protocol. After treatment with IS and PCS, each sample (5×10^6^ cells) was analyzed during incubation in a magnetically stirred chamber over a period of 5 min at constant temperature (37 °C). The signals were detected and analyzed using built-in software from Strathkelvin Instruments. The rate of oxygen consumption was normalized to the number of living cells, which was determined using by the Cellometer Vision CBA image cytometer (Nexcelon, Lawrence, MA).Three wells were utilized per condition in any given experiment.

### Mitochondrial membrane potential

The mitochondrial membrane potential was examined using the JC-1mitochondrial potential sensor (Invitrogen, Carlsbad, CA). After treatment with IS and PCS, cells were incubated with the JC-1 dye (10 μg/mL) at 37°C for 15 min, followed by analysis with the Gallios flow cytometer (Beckman Coulter, Brea, CA) for quantifying 488-nm-excited fluorescence signals at 585/42 nm (FL2; red) and 525/50 nm (FL1; green). JC-1 monomers emit at 530 ±15 nm (FL1 channel), and J-aggregates emit at 590 ± 17.5 nm (FL2 channel). Cytometry settings were optimized for green (FL1) and red (FL2) fluorescence, and the data were analyzed with the Kaluza Flow Cytometry analysis software V1.2 (Beckman Coulter). The relative red to green fluorescence ratio of cells was calculated.

### Glucose uptake and metabolite analysis

The glucose concentration of the culture medium was detected using a glucose meter (Abbott, Chicago, IL). Glucose uptake was calculated by detecting the decrease in the glucose concentrations of the culture medium after IS and PCS treatment. Sample processing and metabolite analysis were performed as previously described [38, 39].

For extracting hydrophilic metabolites, the cells were washed twice with PBS and scraped on ice in 1mL of 80% methanol. The cell lysate was centrifuged at 14,000×gfor 15min at 4°C, and the supernatant was collected for rapid resolution liquid chromatography–time-of-flight-mass spectrometry analysis. A modified Folch's method was employed for extracting lipids. Liquid chromatographic separation for processed cell samples was achieved on a 100× 2.1 mm Acquity 1.7-μm C8 column (Waters Corp., Milford, MA) by using an ACQUITY TM UPLC system (Waters Corp.). The eluent was analyzed using high-definition, time-of-flight mass spectrometry (SYNAPT G1, Waters Corp.) operating in the electrospray ionization-positive ion mode. Raw mass spectrometric data were processed using Mass Lynx V4.1 and Marker Lynx software (Waters Corp.). The multivariate data matrix was analyzed using SIMCA-P software (version 13.0, Umetrics AB, Umea, Sweden).

### Statistical analyses

All data are expressed as mean ± standard error. One-way analysis of variance with Bonferroni corrections was performed for analyzing the data of the cell culture study. The data of the different animal groups were compared using the Wilcoxon–Mann–Whitney test. *P-*values of <0.05 were considered statistically significant.

## SUPPLEMENTARY MATERIALS FIGURES AND TABLES





## Abbreviations

CKD: chronic kidney disease
ROS: reactive oxygen species
TGF: transforming growth factor
PCS: *p*-cresol sulfate
NADPH: nicotinamide adenine dinucleotide phosphate hydrogen
RAAS: renin–angiotension–aldosterone system
DNA: deoxyribonucleic acid
Cisd2: CDGSH iron sulfur domain 2
NNT: nicotinamide nucleotide transhydrogenase
Mfn: mitofusin
LAMP1: lysosomal-associated membrane protein 1
NAC: *N*-acetylcysteine
PAS: periodic acid–Schiff
HUVECs: human umbilical vein endothelial cells
PBS: phosphate-buffered saline
