# Supplementary material for: Protein-bound uremic toxins impaired mitochondrial dynamics and functions
Source: Oncotarget. 2017 Sep 8;8(44):77722–33. doi: 10.18632/oncotarget.20773 (PMC5652810; doi:10.18632/oncotarget.20773)
Supplement: Supplementary file 1 [file oncotarget-08-77722-s001.pdf]

Protein-bound uremic toxins impaired mitochondrial dynamics and functions

SUPPLEMENTARY MATERIALS

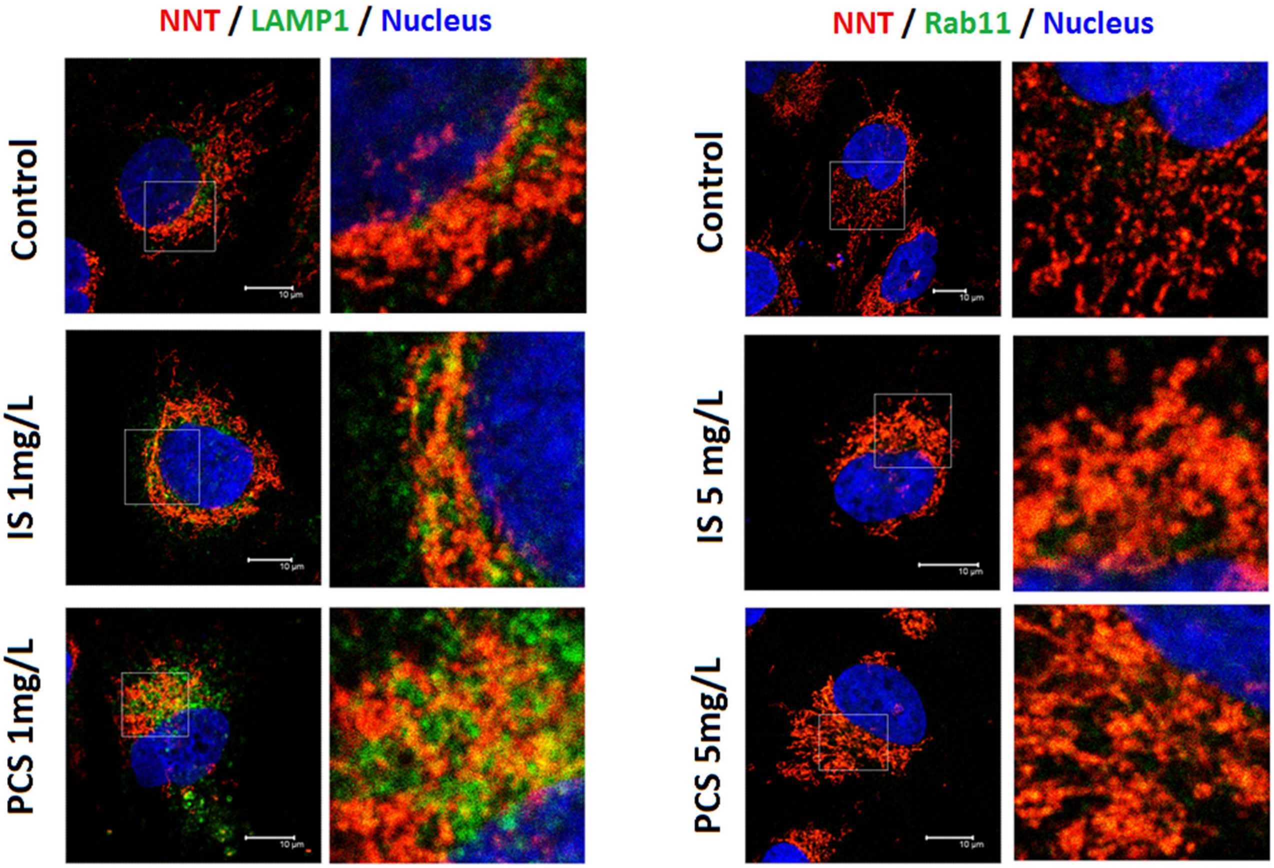

Supplementary Figure 1: Indoxyl sulfate and *p*-cresol sulfate increased mitochondrial localization of LAMP1 and Rab11 *in vitro*. Cultured human renal tubular cells (HK2) treated with IS or PCS under the serum-free condition for 24 were analyzed. Control cells were cultured under the serum-free condition only. The concentrations of IS and PCS for this study are indicated in the figures. The representative results of immunofleurocent staining for LAMP1/NNT and Rab11/NNT were demonstrated.

Supplementary Table 1: Lists of antibodies for western blot, immune-histological and immune-florescent staining

See Supplementary File 1
