# Supplementary material for: Protein-bound uremic toxins impaired mitochondrial dynamics and functions
Source: Oncotarget. 2017 Sep 8;8(44):77722–33. doi: 10.18632/oncotarget.20773 (PMC5652810; doi:10.18632/oncotarget.20773)
Supplement: Supplementary file 2 [file oncotarget-08-77722-s002.docx]

**Supplementary Table 1. Lists of antibodies for Western blot, immune-histological and immune-florescent staining**

| **Antibody** | **Source** | **Western blot** | **Immune-**  **florescent staining** | **Immune-**  **histological staining** | **Company** | **Product number** |
| --- | --- | --- | --- | --- | --- | --- |
| NNT | Mouse | 1:700 | 1:200 |  | Abcam, Cambridge, UK | ab110352 |
| mitofusin-1 | Mouse | 1:500 | 1:100 | 1:500 | Abcam | ab57602 |
| mitofusin-2 | Rabbit | 1:1000 | 1:50 | 1:400 | Cell Signaling, Danvers, MA | 9482 |
| Fis1 | Rabbit | 1:200 |  | 1:50 | Sigma, Victoria, Australia | HPA017430 |
| LC3B | Rabbit | 1:1000 | 1:400 |  | Cell Signaling, | 2775 |
| Cisd2 | Rabbit | 1:1000 | 1:50 | 1:400 | Thermo Fisher,  Waltham, MA | PA5-34545 |
| Beta-actin | Mouse | 1:5000 |  |  | Cell Signaling | 3700 |
| LAMP1 | Rabbit | 1:1000 | 1:1000 | 1:200 | Abcam | ab24170 |
| phospho-Drp1 | Rabbit | 1:1000 | 1:400 |  | Cell Signaling | 3455 |
| Drp1 | Rabbit | 1:1000 | 1:50 |  | Cell Signaling | 8570 |
| Rab11 | Rat |  | 1:100 |  | Abcam | Ab 180504 |
| Parkin | Rabbit | 1:500 | 1:50 | 1:50 | Abgnet, Shanghai, China | AP6402a |
| GFP | Rabbit | 1:1000 |  |  | Abcam, | ab290 |
| Alexa Fluor 594 Chicken Anti-Mouse IgG | Chicken |  | 1:1000 |  | Thermo Fisher | A21201 |
| Alexa Fluor 488 Chicken Anti-Rabbit IgG | Chicken |  | 1:1000 |  | Thermo Fisher | A21441 |

(NNT: nicotinamide nucleotide transhydrogenase; Fis1: mitochondrial fission 1; LC3B: microtubule-associated protein 1 light chain 3 beta; Cisd2: CDGSH iron sulfur domain 2; LAMP1: lysosomal-associated membrane protein 1; Drp1: Dynamin-1-like protein)
